# Analysis of Entrepreneurial Motivation on Entrepreneurial Psychology in the Context of Transition Economy

**DOI:** 10.3389/fpsyg.2021.680296

**Published:** 2021-08-11

**Authors:** Baisheng Shi, Tao Wang

**Affiliations:** ^1^School of Economics and Management, Wuhan University, Wuhan, China; ^2^Research Center for Organizational Marketing of Wuhan University, Wuhan, China

**Keywords:** transition economy, entrepreneurial motivation, entrepreneurial psychology, entrepreneurial environment, social psychology

## Abstract

This study aims to explore the connotation of entrepreneurial motivation in the context of a transitional economy. The actual situations of 135 young entrepreneurs are investigated based on questionnaire surveys and case analyses. The influences of different entrepreneurial motivation factors on entrepreneurial psychology are analyzed. Results demonstrate that the item of “gain wealth” on the entrepreneurial motivation scale has the highest score of 3.98 points. In contrast, the scores of opportunity motivation and survival motivation are basically the same, with great differences between different individuals. The dimensions of policies, regulations, and government functions on the entrepreneurial environment scale have high ranks and levels, while the support of industry associations needs to be strengthened. Generally, the entrepreneurial psychology scale has high scores; most of the items score above 3.45 points, indicating that entrepreneurship not only improves the quality of work and life for young entrepreneurs but also promotes the personal growth of entrepreneurs. The multiple stepwise regression analysis reveals significant correlations between survival and opportunity motivations of young entrepreneurs and their subjective psychology and social psychology. The results can provide experimental and useful reference materials for the following investigations.

## Introduction

The world has gradually entered the knowledge economy era that depends on high-tech and innovative talents and regards the development of science and technology as the driving force to promote the sustained and rapid development of the social economy (Thurik et al., 2013; Wu and Song, 2019). At the same time, entrepreneurship can further improve social stability and promote the sustained and rapid development of the economy. Thus, the government also focuses on entrepreneurship. At the current stage, the concept of “Entrepreneurship by All and Innovation by All” has been deeply rooted in the hearts of the people driven by the market environment and national policies. Entrepreneurship has become a consensus of the public and a key issue that local governments at all levels focus on (Avgerou and Li, 2013; He et al., 2019). China has launched rich and colorful entrepreneurship education programs and projects for young people, introducing them to new entrepreneurial fields, providing them with more entrepreneurial opportunities, helping them build a broader entrepreneurial platform, and teaching them knowledge about entrepreneurial projects (Su et al., 2015; Yuan et al., 2020). However, policy support is far from enough. Due to the lack of education on entrepreneurial psychology in entrepreneurship, young people are prone to psychological fluctuations and mood swings, leading to the loss of confidence in entrepreneurship. What is worse, some even choose to give up in the middle of entrepreneurship. Therefore, it is necessary to strengthen and analyze the entrepreneurial psychology of youth groups objectively and practically.

At the current stage, entrepreneurial psychology analysis for young people generally focuses on entrepreneurial psychology education for college students (Sieja and Wach, 2019). Ng et al. (2016) investigated the basic characteristics and rules of the use of micro-business of undergraduates to start a business and analyzed the feminine characteristics of micro-business. Kyoung and Il-Han (2016) studied the impact of creativity and self-efficacy of college students on entrepreneurial intentions and analyzed the internal connections of various factors. Young entrepreneurs of China are the backbone of its social and economic development. Entrepreneurs in the new era have distinct characteristics of the times, which have adequately promoted the economic and social development of China. Therefore, how the entrepreneurial motivation of Chinese entrepreneurs changes, how the uncertainty of the transition economy environment affects the entrepreneurial psychology of young people, and how youth entrepreneurship survive and develop are the practical problems currently affecting the social entrepreneurial atmosphere, entrepreneurial economic development, and the mental health of youth entrepreneurship of China. These are also key issues that deserve attention. At the current stage, studies on young entrepreneurial psychology consider college students as research objects rather than young entrepreneurs. Meanwhile, entrepreneurial psychology is not distinguished carefully. Therefore, detailed and sufficient research on the entrepreneurial psychology of young entrepreneurs is very necessary.

Based on the basic characteristics of the transition economy of China at this stage, the connotation, entrepreneurial motivation, and entrepreneurial motives of young entrepreneurs are studied profoundly through theoretical research and empirical analysis. Methods adopted include questionnaire surveys and interviews, aiming to explore the fundamental situation, the entrepreneurial motivation, and the entrepreneurial psychology of young entrepreneurs. Besides, the transition economy environment at this stage is analyzed to comprehensively explore the influence of entrepreneurial motivation on entrepreneurial psychology. Results demonstrate that entrepreneurial behavior has significantly improved the quality of life of young entrepreneurs. The opportunity-oriented and psychological-oriented differences between different entrepreneurs are apparent. Relevant industries need to strengthen their support for young entrepreneurs further. In summary, the above results can provide scientific and effective reference materials for the subsequent analysis of entrepreneurial psychological factors of young entrepreneurs.

## Literature Review

### Current Economic Background of China

The environmental changes during the transition economy stage have a significant influence on the rationality of entrepreneurial behavior and the development of entrepreneurial activities (Boso et al., 2013; Sun and Xu, 2017). Chinese society is undergoing tremendous changes in the context of the transition economy. These changes will profoundly impact the strategic choices of enterprises and also bring entrepreneurial opportunities and challenges to Chinese enterprises and organizations that need strategic transformations (Mazzucato, 2015). Under such an environment, various Chinese enterprises have begun to adopt entrepreneurial-oriented strategies, which also put forward new requirements for the abilities and psychological states of young entrepreneurs (Guo and Jiang, 2013).

The entrepreneurial environment can be divided into the entrepreneurial government policy environment, the entrepreneurial market business environment, and the entrepreneurial social and cultural environment (He et al., 2020). The government policy environment for entrepreneurship includes entrepreneurial policies, tax policies, financial support, and research and developmental support. The market business environment for entrepreneurship includes business environment and infrastructure construction, intellectual property protection, and markets. The social and cultural environment for entrepreneurship includes social atmosphere, cultural environment, and social norms (Malebana, 2014). However, the government policy and the market business environments have significant turbulence, uncertainty, and discontinuity. Therefore, the entrepreneurial environmental risks and challenges faced by young entrepreneurs are derived from the changing government policies and market policies. On the one hand, policy changes in the entrepreneurial environment may bring unexpected changes to the business operations of young entrepreneurs, resulting in restrictions and hindrances to the development of the enterprise, and ultimately, putting pressure on the psychology of young entrepreneurs (Siu and Lo, 2013). On the other hand, changes in government and market policies often contain huge business opportunities. Young entrepreneurs who are forward-looking and good at using policy advantages can obtain favorable opportunities and support, which play a decisive role in the survival and development of their enterprises, thereby promoting their entrepreneurial success.

### Literature Review on Entrepreneurial Psychology

At this stage, research on entrepreneurial psychology is concentrated on college students and entrepreneurs. Most universities worldwide vigorously carry out innovation and entrepreneurship education, resulting in numerous studies on the entrepreneurial psychology of college students (Li and Wu, 2019; Liu et al., 2019). In contrast, research on entrepreneurial psychology is rarely reported. Saseendran and Salman (2019) took entrepreneurs of small businesses as research objects and explored the correlation between entrepreneurial psychology and small business management. Fadzil et al. (2019) took Malaysian e-commerce practitioners as research objects and explored the correlation between the entrepreneurial psychology of e-commerce entrepreneurs and entrepreneurial ability. Feng and Chen (2020) suggested that entrepreneurial passion played an essential role in entrepreneurial spirit and entrepreneurial behavior. The above studies reveal that most studies on entrepreneurship focus on the same industry or the same group. Moreover, most studies explore only one of the various factors involved in entrepreneurship. Hence, it is necessary to focus on the specific connotation and definition of entrepreneurship and comprehensively consider entrepreneurship.

### Literature Review on Entrepreneurial Motivation

The pursuit of wealth has always been considered the most critical component of entrepreneurial motivation. As the research goes deeper, more researchers have noted other aspects of entrepreneurial motivation, analyzing how various factors affect entrepreneurial motivation from psychology. Su et al. (2020) investigated the information in the social accounts of six entrepreneurs and explored the interaction between positive emotions and entrepreneurial motivation. Results suggested that positive emotions could affect entrepreneurial motivation and entrepreneurial process of entrepreneurs significantly. Bartha et al. (2019) examined the entrepreneurial motivation of entrepreneurs in Central and Eastern Europe and explained that social mission factors were the main factors affecting the entrepreneurial motivation of entrepreneurs. Rajabi et al. (2018) explored the relationship between entrepreneurial motivation and enterprise development in the sales industry, indicating that entrepreneurial motivation positively affected the development of enterprises (Hemmert et al., 2019).

The above studies suggest that both entrepreneurial psychology and entrepreneurial motivation of entrepreneurs are significantly influencing factors in the entrepreneurial process, which are directly linked to the development and operation of enterprises. Therefore, entrepreneurial psychology and entrepreneurial motivation are critical, which are the exploration and analysis target in this study. However, most studies have investigated entrepreneurial psychology and entrepreneurial motivation separately, while the impacts of the industry and the surrounding environment of entrepreneurs are rarely considered. Therefore, in this study, the entrepreneurial psychology, entrepreneurial motivation, and entrepreneurial environment are organically combined to explore the *status quo* of entrepreneurial entrepreneurship as comprehensively as possible, making the research results more practical.

## Methodology

### Sample and Procedure

#### Entrepreneurs in the New Era

Theoretically, the group of entrepreneurs that emerged after the Reform and Opening-up of China is generally referred to as “the first generation of entrepreneurs” in the private economy of China. The new generation of entrepreneurs that emerged in the new wave of globalization and industrial upgrading is called “the second generation of entrepreneurs” (Ligtvoet, 2018; Garaika et al., 2019). Compared with the older generation of entrepreneurs, young entrepreneurs have higher education levels and more open access to information; meanwhile, the public opinion attitude of the environment is more relaxed. Compared with “the first generation of entrepreneurs,” the motivation, entrepreneurial skills, and abilities of young entrepreneurs have changed significantly (Manbachi et al., 2018). However, under the current transition economy environment in China, the turbulence, uncertainty, and instability of government policies and business environment have exacerbated the complexity of youth entrepreneurship.

At the current stage, the entrepreneurial environment of China is in a stage of active development. Various localities actively launch various activities to support youth groups and actively implement a series of preferential policies to actively encourage youth groups to participate in entrepreneurial activities (Kang et al., 2021). In addition, universities also actively promote innovation and entrepreneurship activities for college students, encouraging students with the entrepreneurial propensity to start entrepreneurial activities while they are in schools, laying a solid foundation for subsequent entrepreneurial activities. In summary, at this stage, the entrepreneurial environment of Chinese society for young people has been improving, showing a good developmental trend. However, due to regional differences and gaps in entrepreneurial industries, entrepreneurial policies in different industries and industries in the transition economy stage are numerous, change rapidly, and have uneven standards, lacking continuity and stability. In the meantime, different regional entrepreneurial environments in China are very different, and the intensity and tendency of government policies and services are quite different as well. Therefore, governments must continue to increase their support for young entrepreneurs and promote the entrepreneurial activities of young entrepreneurs, providing a significant impact on the development of young entrepreneurs and the entire social environment.

#### Entrepreneurial Psychology

Entrepreneurial psychology can be summarized as the subjective feelings of young entrepreneurs and the overall psychological evaluation of their work performance, quality of life, personal growth, and social identity in the process of establishing and operating a business. At present, there is no clear definition of entrepreneurial psychology (Obschonka et al., 2020). Therefore, entrepreneurial psychology is divided into two dimensions based on theoretical analysis and practical research, namely, entrepreneurial subjective psychology and entrepreneurial social psychology. First, subjective psychology includes the feelings of young entrepreneurs about their work performance, quality of life, and personal growth, including the sense of accomplishment, self-confidence, and life value gained and felt from entrepreneurial activities. Second, social psychology refers to feelings of young entrepreneurs about social identity, including products or services that can help others or promote social progress (Luca, 2017).

#### Entrepreneurial Motivation

Entrepreneurial motivation is the primary link to stimulate the start of entrepreneurship. In the meantime, entrepreneurial motivation can also support young entrepreneurs to engage in entrepreneurial activities and achieve entrepreneurial goals in the subsequent entrepreneurial process, which is regarded as the inner driving force of young entrepreneurs (Barba-Sánchez and Atienza-Sahuquillo, 2017). In this regard, entrepreneurial motivation plays an essential role in entrepreneurial activities and is also the key issue in entrepreneurial research. According to the research results of the *Global Entrepreneurship Observation Report*, entrepreneurial behavior is divided into “survival entrepreneurship” and “opportunistic entrepreneurship” (Ismail et al., 2016). Survival entrepreneurship refers to the non-optional or enforced entrepreneurial activities when someone cannot find a satisfactory job due to personal reasons or cannot get any jobs due to other conditions. Opportunistic entrepreneurship refers to the entrepreneurial activities that someone engages in to pursue personal ideals and values or because of business opportunities discovered in the market (Aima et al., 2020). In this study, the entrepreneurial motivation categories of “survival entrepreneurship” and “opportunistic entrepreneurship” will be referenced. At present, research on entrepreneurs and entrepreneurship of college students is various; however, investigations of entrepreneurial motivation are still in the developmental stage. In this regard, analyzing entrepreneurial motivation and its influence on entrepreneurial psychology will be the focused in this study.

### Measures

The research methods are questionnaire survey and interview. The scoring standard of the survey adopts the five-point Likert scale, that is, 1–5 points correspond to complete non-conformity to complete conformity. Survey samples are selected based on the information of young entrepreneurs registered in alumni associations and provided on social network sites. The questionnaire is distributed online and offline. A total of 180 questionnaires were issued, and 135 valid questionnaires were returned, with a response rate of 75%. The contents of the questionnaire survey and interview are as follows.

#### Content of the Questionnaire Survey

This questionnaire survey scale comprises three subscales, namely, the entrepreneurial motivation orientation scale, the entrepreneurial environment perception scale, and the entrepreneurial psychology scale (Cheng, 2019). Entrepreneurial motivation is divided into two dimensions, namely, opportunity motivation and survival motivation. The opportunity motivation dimension includes three sub-dimensions, namely, “realize dreams,” “prove himself,” and “market opportunities.” The survival motivation dimension also includes three dimensions, namely, “gain wealth,” “dissatisfied with the *status quo*,” and “employment difficulties.” The entrepreneurial motivation scale consists of six questions, which are recorded as S1-1 to S1-6.

The entrepreneurial environment scale is divided into two dimensions, namely, policy environment and business environment (Zhang et al., 2016). The government environment refers to the subjective score based on the degree of identity described in four variables, namely, policies and regulations, government functions, government information, and financing services. Policies and regulations explore whether local governments give priority to supporting newly established and growing enterprises when formulating policies. Government functions analyze whether government functions have changed and adapted to market requirements. Government information investigates whether government departments can provide valuable entrepreneurial projects and information. Financing services reveal whether the government provides good services or subsidies in financing startups. The business environment includes six variables, namely, intellectual property rights, infrastructure, technical services, product import and export conditions, market services, and industry association support. These variables successively analyze whether intellectual property rights, such as trademarks and patented technologies, can be protected when young entrepreneurs start businesses, whether infrastructure, such as water supply, power supply, transportation, and communication, is affordable, whether high-level technical support is available, whether good export conditions are available in the local area, whether services, such as accountants and lawyers, are convenient, and whether aids can be easily obtained from local industry associations. This scale has 10 questions, denoted as S2-1 to S2-10.

The entrepreneurial psychology scale includes two dimensions, namely, entrepreneurial subjective psychology and entrepreneurial social psychology (Yueh et al., 2020). Variables of entrepreneurship subjective psychology include work quality perception, life quality perception, and personal growth perception; the principal variable of entrepreneurial social psychology is social identity perception. Work quality perception analyzes work performance, business operations, and work status of young entrepreneurs; life quality perception investigates off-work entertainments, physical condition, and current life status of young entrepreneurs; personal growth describes entrepreneurship achievement, personal self-confidence, and the accomplishment of life values through entrepreneurship. The social identity perception is analyzed from the perspective of entrepreneurship helping others, promoting social progress, and improving social status. The scale comprises a total of four questions, which are denoted as S3-1 to S3-4, respectively.

**FIGURE 1:**
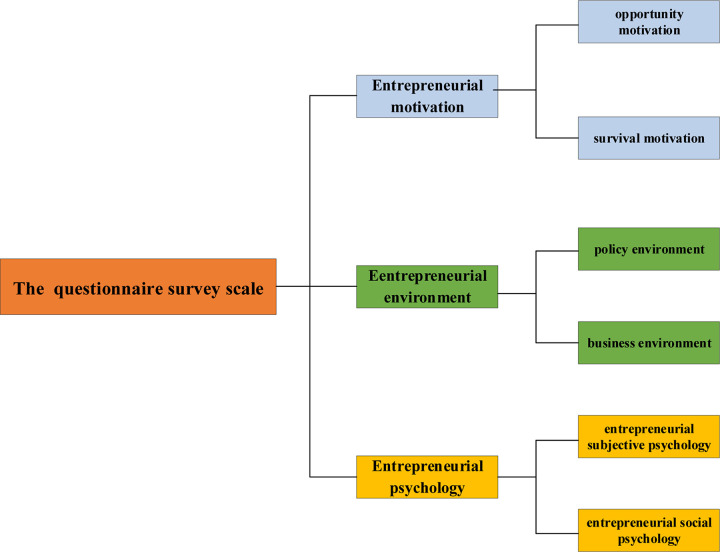
Overview of the questionnaire survey.

The overall questionnaire survey is shown in Figure 1.

### Survey Sample Selection

The interview is targeted, focusing on the influence of entrepreneurial motivation on entrepreneurial psychology. The interviewees are 10 young entrepreneurs of different genders from different industries. The information of surveyed entrepreneurs comes from the alumni association of the school and social media. The research samples are young entrepreneurs under the age of 40, who are one of the founders of their enterprises and have a high degree of investment in the entire entrepreneurial activity process. They are familiar with the operating conditions of enterprises and the external environment.

## Research Results

### Questionnaire Survey and Statistical Analysis of Research Samples

#### Statistical Analysis of Research Samples

**FIGURE 2:**
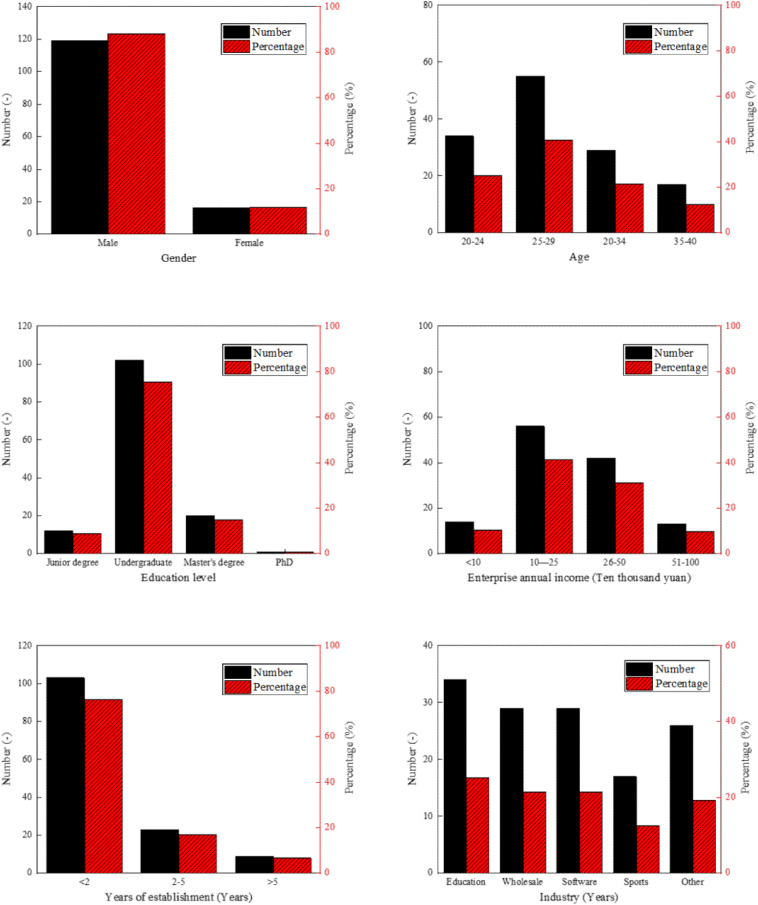
Summary of basic information of research samples.

First, statistical analysis is performed on the basic information of the gender, age, education, annual income, enterprise establishment time, and industry of the samples. The results are shown in Figure 2.

As shown in Figure 2, among the surveyed entrepreneurs, 119 are male entrepreneurs, accounting for 88.1%; only 16 are female entrepreneurs, accounting for 11.9%. Moreover, those aged 25–29 years account for the majority (55 people, accounting for 40.7% of the total). The number of entrepreneurs with a bachelor’s degree is the largest, followed by those with a master’s degree; those with a doctoral degree or graduated from vocational colleges account for the minority. Among the enterprises surveyed, the number of enterprises with an annual income of 100,000–500,000 Chinese yuan is the largest, accounting for 72.5% of the total enterprises. Most enterprises have been established for less than 2 years. The industries that the surveyed enterprises are engaged in include education, wholesale and retail, software information, cultural, sports, and entertainment, and others. The above survey results suggest that among the young entrepreneurs surveyed, the number of male entrepreneurs is significantly higher than that of female entrepreneurs. The entrepreneurs are of lower ages and higher educational backgrounds. The surveyed enterprises have a short establishment time and are widely distributed in various industries.

#### Analysis of the Scale

**FIGURE 3:**
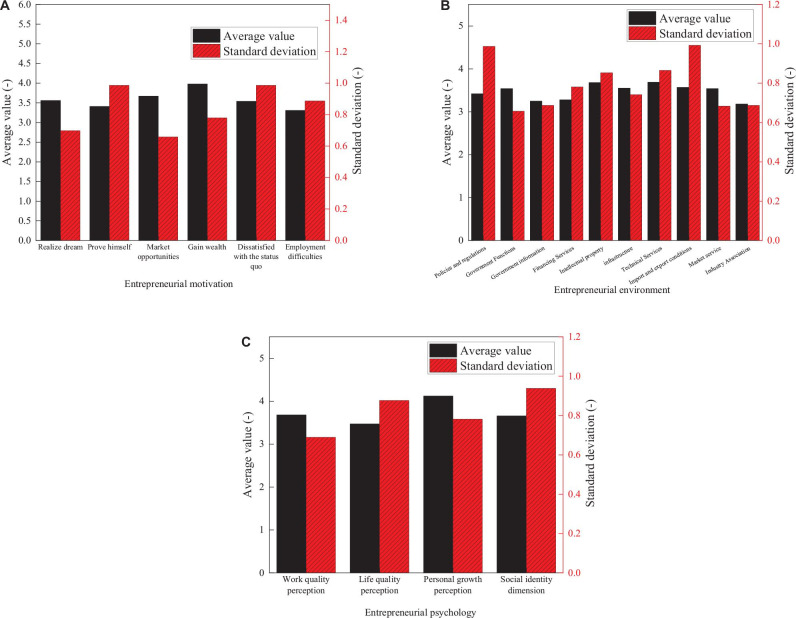
Scores of questionnaire survey results. **(A–C)** represent the entrepreneurship motivation scale, entrepreneurship environment scale and entrepreneurship psychology scale respectively.

In the meantime, the survey results of the entrepreneurial motivation scale, the entrepreneurial environment scale, and the entrepreneurial psychology scale are analyzed. The scores of these three scales are presented in Figures 3A–C.

As shown in Figure 3, the item of “gain wealth” on the entrepreneurial motivation scale has the highest score, 3.98 points. The motivation of employment difficulties has the lowest score, only 3.31 points. In this survey, the scores of opportunity motivation and survival motivation are basically the same. However, the SD of the survival motivation dimension is obviously greater than that of the opportunity motivation dimension. Meanwhile, the entrepreneurial motivation of individual young entrepreneurs is not a single but multiple coexistences. Generally, there are 2–3 most prominent motivations. The surveyed young entrepreneurs have diverse entrepreneurial motivations, with great differences between different individuals.

The technical support dimension and intellectual property dimension on the entrepreneurial environment scale have the highest scores, which are 3.69 and 3.68 points, respectively. In the meantime, the scores of policies and regulations and government function dimensions are also around 3.5 points, indicating that policy support is strong at the current stage. The dimension with the lowest score is the industry association support. The reason is that most enterprises are in the developmental stage. The support of industry associations is insufficient, which is also a point of concern for future development.

Scores of the entrepreneurial psychology scale are generally higher than the other two scales. Most of the items score above 3.45 points. The “personal growth” dimension has the highest score, 4.12 points; the quality of life dimension has the lowest score, 3.47 points. The survey results prove that entrepreneurship not only brings improvements in work and life quality for young entrepreneurs; more importantly, it brings tremendous growth to young entrepreneurs.

In the meantime, 10 young entrepreneurs are interviewed. The results show that as the startup time prolongs and the startup stage changes, the psychology of different young entrepreneurs changes. Their entrepreneurial motivation will also change accordingly. In the initial stage, developmental stage, and maturity stage, the motives and goals of young entrepreneurs will change in categories or increase or decrease in degree.

### Sample Reliability and Validity

**FIGURE 4:**
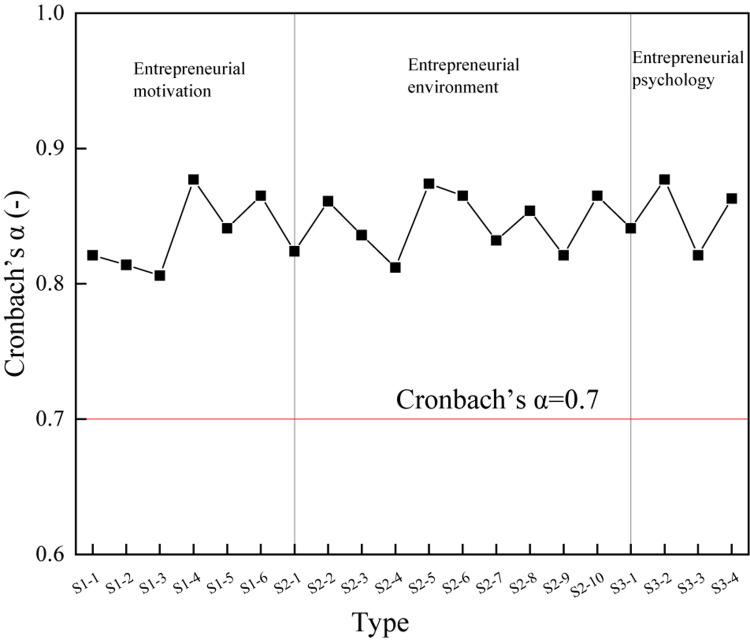
The calculation results of Cronbach’s α coefficient on the scale.

The reliability of the scale is measured by Cronbach’s α coefficient. Generally, the value of Cronbach’s α coefficient is required to be > 0.7; the closer to 1, the better the effect. The Cronbach’s α coefficient of each item is calculated by the SPSS 22.0 (IBM, New York, United States) software, and the results are shown in Figure 4.

As shown in Figure 4, the Cronbach’s α coefficient value for each item of all the three scales is > 0.7, indicating that the scale is very reliable and meets the reliability requirements.

The scale validity test calculates the Kaiser–Meyer–Olkin (KMO) values and Bartlett sphere test observations. The specific results are shown in Table 1.

**TABLE 1:** KMO and Bartlett sphere test results of three scales.

**Type**	**KMO**	**Approx. Chi-Square**	**df**	***P***
Entrepreneurial motivation	0.903	1098.25	29	0.000
Entrepreneurial environment	0.854	698.54	78	0.000
Entrepreneurial psychology	0.874	874.25	56	0.000

As shown in Table 1, the KMO values of the entrepreneurial motivation scale, the entrepreneurial environment scale, and the entrepreneurial psychology scale are 0.903, 0.854, and 0.874, respectively, all > 0.8. The Bartlett sphere test observations are 1,098.25, 698.54, and 874.25; besides, all the *P* values are 0.000, less than the given significance level of 0.01, indicating that the variables are correlated and have passed the validity test.

The above analyses suggest that all the questions involved meet the requirements of reliability and validity. Hence, all questions can be subjected to follow-up analysis.

### Analysis of Survey Results

The multiple stepwise regression analysis is adopted. The control variables include the gender, education, and the industry of young entrepreneurs to avoid the influence of other factors on the principal effect. During calculations, the education variable is divided into four categories: vocational college, bachelor (undergraduate), master (graduate), and doctor, with values 1–4 points in order. A gender variable of 1 represents male and 0 represents female. The industries of the surveyed enterprises are classified into five categories, namely, education, wholesale and retail, software and information, cultural, sports, and entertainment, and others. The correlation coefficients are calculated, and the specific results are summarized in Table 2.

**TABLE 2:** Correlation coefficients.

	**Gender**	**Education**	**Industry**	**Opportunity motivation**	**Survival motivation**	**Entrepreneurial subjective psychology**	**Entrepreneurial social psychology**
Gender	1						
Education	0.094	1					
Industry	0.105	0.903	1				
Opportunity motivation	0.125	0.121	−0.031	1			
Survival motivation	0.098	0.097	0.088	0.532***	1		
Entrepreneurial subjective psychology	0.103	0.993	0.108	0.598***	0.474***	1	
Entrepreneurial social psychology	0.112	−0.117	0.117	0.347***	0.339***	0.238***	1

***indicate *P* < 0.01.

As shown in Table 2, the gender, education, and industry of the entrepreneur are not significantly associated with survival motivation, opportunity motivation, entrepreneurial subjective psychology, and entrepreneurial social psychology of young entrepreneurs. Thus, it proves that the gender, education, and industry of the entrepreneur will not significantly impact the entrepreneurial psychology and entrepreneurial motivation of the entrepreneur. The correlation coefficients of opportunity motivation to survival motivation, entrepreneurial subjective psychology, and entrepreneurial social psychology are 0.532, 0.598, and 0.347, respectively. The correlation coefficients of survival motivation to entrepreneurial subjective psychology and entrepreneurial social psychology are 0.474 and 0.339, respectively. The correlation coefficient between entrepreneurial subjective psychology and entrepreneurial social psychology is 0.238.

## Discussion

Analyzing and summarizing the questionnaire survey results can reveal the basic situations of the entrepreneurs surveyed and the correlation between entrepreneurial motivation and entrepreneurial psychology.

According to the basic *status quo* of entrepreneurs, with the development and progress of society, most entrepreneurs have a higher educational background and are well-educated. Compared with the previous generation of entrepreneurs, the knowledge level of entrepreneurs in the transitional economy of the new era is higher, conducive to using scientific management methods for enterprise management. Simultaneously, subjected to various restrictions, most entrepreneurs surveyed are males, while females account for a small proportion. This may be caused by the number of surveyed samples and the surveyed industry. With the innovation and entrepreneurship policies of China, most entrepreneurs are younger, having a lot of energy and enthusiasm for work, which is very conducive to enterprise development. The education industry has the largest number of entrepreneurs. This is because the most common study for young entrepreneurs during school is the education industry, the industry that is familiar to them. Thus, many students are engaged in this industry after graduation.

Regarding entrepreneurial motivation, acquiring wealth is the main factor for entrepreneurs to start a business; however, acquiring wealth is not the only factor for entrepreneurs to start a business, and most entrepreneurial motivation of entrepreneurs is diversified. In the meantime, with the progress of the times and economic development, the differences between different entrepreneurs have gradually increased, and the entrepreneurial motivation of everyone shows increasing differences. Supported by laws, regulations, and policies, entrepreneurs have a better entrepreneurial environment, and the market and laws support greater strength, which is very suitable for entrepreneurial activities. However, industry support is insufficient in the enterprises under investigation. Therefore, in the follow-up development of enterprises, entrepreneurs should fully mobilize the power of the industry, seek industry support, and enable enterprises to achieve better development. Entrepreneurship not only brings economic freedom and wealth to entrepreneurs but also greatly enhances the personal abilities of entrepreneurs, which is of great help to improve the comprehensive capabilities of entrepreneurs.

The results of the model analysis revealed a significant correlation between the survival motivation and opportunity motivation of young entrepreneurs, as well as the personal psychology and social psychology of young entrepreneurs. This proves a significant correlation between entrepreneurial motivation and the psychology of young entrepreneurs. Meanwhile, the entrepreneurial motivation of young entrepreneurs can positively affect entrepreneurial psychology. The opportunity-motivated young entrepreneurs, that is, those who pursue their personal ideals, have higher positive emotions in their entrepreneurial psychology.

Furthermore, the correlation between entrepreneurial motivation and entrepreneurial orientation of young entrepreneurs is analyzed. Compared with the previous study, the actual situation of the transition economy environment at this stage is considered (Zhang and Zhang, 2013). Entrepreneurial motivation and entrepreneurial psychology are divided reasonably, giving more practical reference significance of the results.

## Conclusion

Most entrepreneurs surveyed are males who aged 25–29 years; most entrepreneurs have higher educational backgrounds, and their annual income is about 100,000 to 500,000 Chinese yuan. According to the results of the analysis of the scale items, most entrepreneurs take “gain wealth” as the foremost entrepreneurial motivation. In the meantime, the surveyed young entrepreneurs have diverse entrepreneurial motivations, with two or three prominent motivations, and the differences between different individuals are significant. At the current stage, the entrepreneurial environment of China is comparatively sound. The items of policies, regulations, and government functions score around 3.5 points. Policy support is strong, but the support of industry associations is weak. The personal growth dimension on the entrepreneurial psychology scale has the highest score. The interview results suggest that entrepreneurial motivation and entrepreneurial psychology of young entrepreneurs change over time. The correlation coefficient analysis reveals significant correlations between survival motivation and opportunity motivation of young entrepreneurs and their subjective psychology and social psychology. The positive sentiment in entrepreneurial psychology of opportunity-motivated young entrepreneurs is higher. However, due to the objective limitations, only the situation at this stage in China is analyzed, while the psychological motivation of entrepreneurs in other countries and the specific results under economic crisis conditions are not investigated, which will be the future research directions to improve the comprehensiveness of the results. Moreover, due to sample limitations, there are fewer female entrepreneurs surveyed. Hence, the sample size will be expanded in the future, balancing the male-to-female ratio to increase the practical application value further.

## Data Availability Statement

The original contributions presented in the study are included in the article/supplementary material, further inquiries can be directed to the corresponding author/s.

## Ethics Statement

The studies involving human participants were reviewed and approved by Wuhan University Ethics Committee. The patients/participants provided their written informed consent to participate in this study. Written informed consent was obtained from the individual(s) for the publication of any potentially identifiable images or data included in this article.

## Author Contributions

Both authors listed have made a substantial, direct and intellectual contribution to the work, and approved it for publication.

## Conflict of Interest

The authors declare that the research was conducted in the absence of any commercial or financial relationships that could be construed as a potential conflict of interest.

## Publisher’s Note

All claims expressed in this article are solely those of the authors and do not necessarily represent those of their affiliated organizations, or those of the publisher, the editors and the reviewers. Any product that may be evaluated in this article, or claim that may be made by its manufacturer, is not guaranteed or endorsed by the publisher.
